# Serum midkine as a surrogate biomarker for metastatic prediction in differentiated thyroid cancer patients with positive thyroglobulin antibody

**DOI:** 10.1038/srep43516

**Published:** 2017-02-27

**Authors:** Qiang Jia, Zhaowei Meng, Ke Xu, Xianghui He, Jian Tan, Guizhi Zhang, Xue Li, Na Liu, Tianpeng Hu, Pingping Zhou, Sen Wang, Arun Upadhyaya, Xiaoxia Liu, Huiying Wang, Chunmei Zhang

**Affiliations:** 1 Department of Nuclear Medicine, Tianjin Medical University General Hospital, Tianjin, P.R. China; 2 Tianjin Key Laboratory of Lung Cancer Metastasis and Tumor Micro-environment, Tianjin Lung Cancer Institute, Tianjin Medical University General Hospital, Tianjin, P.R. China; 3 Department of General Surgery, Tianjin Medical University General Hospital, Tianjin, P.R. China

## Abstract

Serum thyroglobulin (Tg) is the main post-operative tumor biomarker for patients with differentiated thyroid cancer (DTC). However, the presence of thyroglobulin antibodies (TgAb) can interfere with Tg level and invalidate the test. In this study, we aimed to investigate the predicative value of midkine (MK) as a cancer biomarker for DTC patients with positive TgAb before the first ^131^I therapy. MK levels were measured by enzyme-linked immunosorbent assay in 151 recruited DTC patients after exercising strict inclusion and exclusion criteria. There were 28 TgAb positive DTC patients with metastases and 123 DTC patients without metastases. The value of pre-^131^I-ablative MK to predict metastasis was assessed by receiver operating characteristic (ROC) curves in these two groups of patients. MK levels in the TgAb positive DTC patients were significantly higher than the DTC patients without metastases. ROC showed good predictability of MK, with an area under the curve of 0.856 (P < 0.001), and a diagnostic accuracy of 83% at the optimal cut-off value of 550 pg/ml. In conclusion, we show that MK can potentially be used as a surrogate biomarker for predicting DTC metastases when Tg is not suitable due to TgAb positivity.

Serum thyroglobulin (Tg) is generally regarded as the mainstay post-operative tumor biomarker for patients with differentiated thyroid cancer (DTC)^1^, ever since its first discovery in 1970s^2^. The fundamental role of Tg for monitoring DTC requires high-quality Tg assays. However, a major problem of Tg assays is the interference of thyroglobulin antibody (TgAb). The currently most commonly used immunoradiometric assay (IMA) for TgAb underestimates serum Tg level, presumably because the endogenous Tgs bound with TgAbs cannot interact with the assay antibodies^3^. The traditional radioimmunoassay (RIA) can either underestimate or overestimate Tg level depending on the assay antibodies^4^,^5^, but RIA is reported to be less prone to the influence of TgAb than IMA^6^. The newly developed liquid chromatography-tandem mass spectrometry (LC-MS/MS) method shows some resistance of TgAb interference^7^,^8^. However, the use of RIA and LC-MS/MS is limited by their availability, the lack of automation, as well as the high cost of LC-MS/MS^3^,^9^,^10^.

The false Tg result can have serious consequences for the follow-up of DTC patients, because it masks the disease status^11^. Nevertheless, the prevalence of TgAb in DTC patients has been reported to vary between 8% to 36%, nearly two-fold higher than in the general population^10^. So, it is mandatory that TgAb should be measured in all specimens sent for Tg testing^1^. Several attempts have been made to address this issue. For instance, Aras *et al*.^12^ showed that combined Tg and TgAb measurements were more informative than Tg only for recurrent and persistent DTC patients. In addition, serial measurements and sequential changes of TgAb have been proposed as a surrogate for Tg to predict disease prognosis^10^,^13^. However, this value of TgAb is not unanimously accepted. For instance, Smooke-Praw *et al*.^14^ showed that TgAb level was not suitable to predict disease progression, recurrence or metastasis. Gorges *et al*.^15^ also demonstrated the same results. Asa *et al*.^16^ reported that ^18^F-FDG PET/CT could be an effective alternative in the detection of recurrence or metastasis, and to resolve the TgAb controversy for such patients. The authors argued that TgAb level should be regarded as related with the immune system rather than the tumor load^14^,^16^. In addition, the reliability of TgAb can also be greatly hampered by the low degree of correlation of commercially available TgAb assays^11^,^17^,^18^. Therefore, DTC patients with undetectable Tg and coexistent TgAb remain a challenge because of the difficulty in determining their clinical situation.

Midkine (MK) is a pleiotropic growth factor prominently expressed during embryogenesis yet down-regulated to a low level in healthy adults. In various pathologies, most notably in cancer, strikingly enhanced MK over-expression has been reported^19^. Besides, since it is a soluble cytokine, serum MK is readily apparent in the blood circulation, making it a relatively convenient and non-invasive biomarker. In fact, the first diagnostic test that quantifies MK is receiving regulatory clearance and entering the clinic^19^. In DTC, three immunohistochemistry studies^20^,^21^,^22^ demonstrated that papillary thyroid cancer (PTC) strongly expressed MK, and MK correlated with PTC clinicopathological features as well as synchronous metastases. Recently, we discovered that MK can potentially be used to screen patients with thyroid nodules for DTC before surgery, and to predict whether metastases exist before ^131^I ablative therapy^23^. So, it is interesting to determine whether MK could be an alternative biomarker for DTC patients with positive TgAb.

In this study, we aimed to evaluate the predictive value of serum MK for DTC patients with positive TgAb before the first ^131^I ablative therapy.

## Results

### Patients’ recruitment

In our analysis cohort, we had 28 TgAb positive DTC patients with metastases (group 1), and 123 without metastases (group 2). All 151 patients were diagnosed with PTC. During the recruitment period, we had a total number of 849 DTC patients in the MK clinical investigation, among them 211 patients had positive TgAb. The prevalence of DTC patients with positive TgAb in our cohort was 24.85% (211/849). However, after implementing exclusion criteria, 60 cases were not included in the current analysis. Specifically, there were 5 cases with other malignancies (3 breast cancer, 2 lung cancer), 22 cases of ischemic diseases (14 myocardial ischemia, 3 myocardial infarction, 4 cerebral ischemia, 1 cerebral infarction), 1 autoimmune diseases (1 multiple sclerosis), 5 kidney diseases (4 diabetic nephropathy, 1 hypertensive nephropathy), 1 neural diseases (1 Alzheimer’s disease), 5 inflammation (5 rheumatoid arthritis), 10 hypertension (including 1 hypertensive nephropathy), 16 diabetes (including 4 diabetic nephropathy).

### Prognostic capabilities of indices

**Figure 1:**
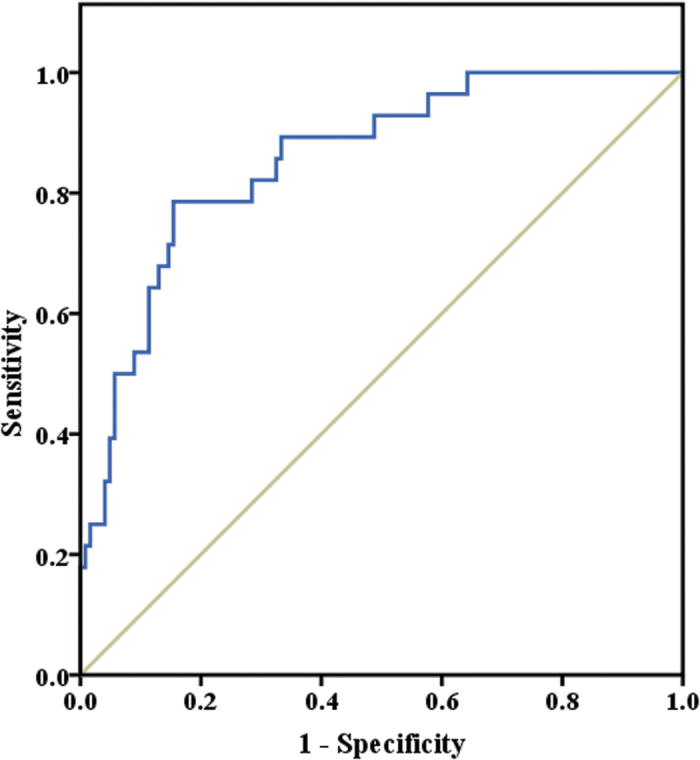
Receiver operating characteristic curves were drawn to determine diagnostic capabilities of pre-^131^I-ablative midkine to discern whether or not metastases existed in patients with differentiated thyroid cancer.

In order to determine the prognostic values, we compared indices of the two groups of patients during the time of their first ^131^I therapy. MK was significantly higher in group 1 than in group 2, while age, TgAb, Tg, free triiodothyronine (FT3), free thyroxine (FT4) and thyroid stimulating hormone (TSH) did not display any significant differences between groups (Table 1). The prognostic capability of pre-ablative MK was conducted by receiver operating characteristic (ROC) curve (Fig. 1), and area under the curves was found to be 0.856 (P < 0.001). Pre-ablative MK showed good ability for predicting metastasis with an optimal cut-off value of 550 pg/ml and a diagnostic accuracy of 83% (Table 2).

## Discussion

Growth factors and cytokines play fundamental roles in various pathological processes. MK is such an important multi-functional heparin-binding growth factor, which regulates cell growth, survival, migration, angiogenic, and anti-apoptotic activities^24^,^25^. MK is rich in both basic amino acids and cysteine^26^, and is the founding member of a small protein family, the other member of which is pleiotrophin^27^,^28^. MK is strongly expressed during embryonic periods, and is crucial in embryonic development, yet its expression in adult tissues is generally very weak. However, MK plays important roles in various pathogenesis, in particular malignant diseases^19^,^25^. MK is generally composed of two domains, namely a more N-terminally located N-domain and a more C-terminally located C-domain^24^,^29^. MK signaling is largely mediated by cell surface receptors like PTPζ^30^. Furthermore, multiple kinases like MAPK and PI3K are important in the downstream signaling system^24^,^25^,^31^. MK over-expression has been reported in diverse oncology settings (for at least 20 different cancer types as pointed out by Jones^19^), making MK a “pan-cancer” biomarker. And opportunities to employ MK as a cancer biomarker exist throughout the disease history of malignancy, from population screening to recurrence monitoring^19^.

The current research focused on MK and thyroid cancer, which is the subject of several previous investigations. An immunohistochemistry study described strongly expressed MK protein in PTC, yet very faint or no expression of MK in normal follicular epithelial cells^20^. Shao *et al*.^21^ reported that strong MK positivity and high expression scores were associated with clinicopathological features of PTC, e.g. extrathyroidal invasion, lymph node metastasis and tumor stages III/IV. Our prior study showed that MK immunohistochemistry could be adopted for differential diagnosis between PTC and multi-nodular goiter, and for prediction of synchronous metastases^22^. Encouraged by the immunohistochemistry results, we performed a serum MK study, evaluating its role as a diagnostic and prognostic biomarker for DTC^23^. We found better diagnostic capability of MK than Tg to differentiate DTC from benign thyroid nodules before surgery. Yet, pre-^131^I-ablative Tg demonstrated a better capability to predict metastases than MK. DTC patients with higher than thresholds MK or Tg levels (500 pg/ml or 20 ng/ml) showed a significantly worse ^131^I-avid metastasis-free survival (Kaplan-Meier method, P < 0.01).

In this investigation, we aimed to assess whether MK could be a surrogate biomarker when Tg was not suitable to monitor the disease due to TgAb positivity in DTC patients. We displayed that MK was a good marker for predicting DTC metastases with an area under the curve value of 0.856 (P < 0.001), and a diagnostic accuracy of 83% with an MK cut-off value of 550 pg/ml. These results further confirmed our previous findings: a diagnostic accuracy of 89% for DTC metastatic prediction with an MK threshold level of 505 pg/ml^23^. The current study also demonstrated that TgAb was not a good predictor, there were no differences in TgAb values between patients with and without metastases, which phenomenon was in accordance with several previous studies^14^,^15^.

In addition to DTC, studies indicate that MK can outperform several currently used blood tumor specific biomarkers, such as alpha fetoprotein (AFP) for hepatocellular carcinoma (HC)^32^,^33^, carcinoembryonic antigen for colorectal cancer^34^, carcinoembryonic antigen and cytokeratin 19 fragments for esophageal squamous cell carcinoma^35^. For instance, Zhu *et al*.^32^ compared MK and AFP in HC tissues and serum samples. The sensitivity of serum MK for HC diagnosis was much higher than AFP (86.9% versus 51.9%) with similar specificities (83.9% versus 86.3%). Notably, serum MK had an outstanding performance in distinguishing AFP-negative HC from control with a sensitivity of 89.2%. Moreover, ROC analysis showed that serum MK had a better performance compared with AFP in distinguishing early-stage HC, small HC, as well as early-stage HC. In another recent study from Shaheen *et al*.^33^, serum MK was significantly elevated in HC compared to cirrhotic and healthy controls. ROC showed that best cutoff for MK and AFP were 0.387 ng/mL and 88.5 ng/mL with areas under the curves of 0.941 and 0.671, respectively. The sensitivity of MK reached 93.3% in patients with AFP < 20 ng/mL.

Although all the above evidence proves that MK can be a sensitive malignant biomarker for DTC, its shortcomings are also obvious, which are also the limitations of the current study. As pointed out by Jones^19^, there are several barriers to overcome before MK can be approved in standard clinical practice. First, larger prospective clinical studies to measure patient outcomes are required to confirm the value of MK, which will allow clinicians to make a better clinical decision leading to a meaningful outcome. Second, elevation of serum MK is not specific to a particular oncology type. We believe the strategy to overcome this limitation is to measure MK in conjunction with other known and specific biomarkers. As we showed in our previous research, diagnostic and prognostic capabilities of MK and Tg should be conducted together^23^. In fact, three multiplex cancer diagnostic tests including MK are currently seeking regulatory approval in the USA. However, the current study indicated that under the circumstance of TgAb influence, neither Tg nor TgAb was appropriate to be used in conjunction with MK, which is a unique situation for thyroid cancer. As described by Jones^19^, depending on the particular clinical circumstance, either measuring MK alone or in combination with other biomarkers could offer utility in certain circumstances. Since all recruited patients in our research were in post-thyroidectomy status with definite DTC pathology, our study on MK was focused on its value for prognosis and follow-up. Third, we want to emphasize, since MK is involved in various diseases, stringent exclusion should be implemented in order to study MK as a biomarker for cancer. In our study, we excluded all patients with other known conditions, although MK levels in some ostensibly healthy cases might still be influenced by unknown conditions. Nevertheless, when patients with a variety of comorbidities are excluded, it would be impossible to known how MK can perform as a tumor marker in DTC patients with such various kinds of co-morbidities. Finally, another important limitation of the study is that MK has been analyzed in patients with PTC after thyroidectomy just before ablation therapy, that is, when they were in a hypothyroid condition. It is unknown about the effect of thyroid hormone on MK levels. It is also unknown how MK performs as a tumor marker in PTC patients when they are treated with thyroid hormone or in patients with follicular thyroid cancer. All these issues will be taken into consideration in our future researching agenda.

## Conclusions

We demonstrate that MK can potentially be used as a surrogate biomarker for the prediction of DTC metastases when Tg is not suitable to monitor the disease due to TgAb positivity. Further longitudinal studies need to be conducted to validate the current finding.

## Patients and Methods

### Patients’ recruitment

Patients’ recruitment protocol was described previously^23^. From January 2011 onward, DTC patients admitted in Nuclear Medicine Department of our hospital awaiting ^131^I treatment were asked to consider entering the MK clinical investigation. If the patients consented, their serum MK levels were measured consequently along with their Tg and TgAb tests during follow-up. For the recruitment of the current investigation, MK database archive retrieval (from January 2011 till December 2015) was performed to identify DTC patients with positive TgAb. Patients with a confirmed post-surgical diagnosis of DTC in pathology and a positive TgAb prior ^131^I therapy were included in the study. Patients with MK influencing co-morbidities such as other malignancies, ischemic diseases, autoimmune diseases, kidney diseases, neural diseases, inflammation, hypertension and diabetes were excluded from the study. Patients with a negative TgAb prior ^131^I therapy were also excluded.

### Treatment and imaging protocols

Management protocol for the DTC patients was conducted generally according to the 2009 guideline from the American Thyroid Association^1^. All DTC patients were given a therapeutic dose of ^131^I for thyroid ablation, after a preparation of thyroid hormone withdrawal. Blood tests were conducted less than 2 days before ^131^I therapy. Routine examinations, including body weight, blood pressure, serum thyroid hormones, blood routine, biochemical indices (liver function, renal function, lipids, glucose), etc., are all measured. Detailed medical histories are collected. Four to 5 days after ^131^I administration, whole body scan (as well as tomography imaging if necessary) was performed by using a dual-detector SPECT/CT machine equipped with high-energy collimators. Before September 2014, Discovery VH SPECT/CT machine (General Electric Medical Systems, Milwaukee Wisconsin, USA) was used for imaging. In September 2014, a new SPECT/CT machine, Discovery NM/CT 670 (General Electric Medical Systems, Milwaukee Wisconsin, USA), was installed and used in our institution. Neck ultrasonography was also performed with frequency range from 3 MHZ to 12 MHZ using PHILIPS HD11 XE (Bothell Washington, USA) to check cervical lymph nodes. Patients were positioned supinely with the neck hyper-extended and imaged by ultrasound. Approximately 6 months after the initial ^131^I therapy, a second ^131^I treatment protocol was implemented to those with confirmed metastases, or a diagnostic ^131^I scan to those without evidence of metastases.

### Diagnosis and follow-up

Final diagnosis was made by our panel of nuclear medicine physicians in consensus, based on a comprehensive consideration of imaging, serological and other clinical materials. All DTC patients were closely followed. After each ^131^I scan was performed, evaluation of each DTC patient was done. For this study, follow-up data were available for at least 6 months after the first ablation for all recruited DTC patients. Metastases were defined as positive hot spots in post-therapeutic ^131^I SPECT/CT scan^36^, all positive cervical lymph nodes were also confirmed by ultrasonography.

### Ethics

The Institutional Review Board of Tianjin Medical University General Hospital approved the ethical and methodological aspects of this investigation. All participants provided their written informed consents to participate in this study. We confirm that all methods were performed in accordance with the relevant guidelines and regulations.

### Serum parameters’ measurement

FT3, FT4 and TSH assays were conducted on a fully automated ADVIA Centaur analyzer (Siemens Healthcare Diagnostics, USA). Tg and TgAb were assessed on a fully automated IMMULITE 2000 analyzer (Siemens Healthcare Diagnostics, USA). These assays were based on chemiluminescent reaction principle.

The calibration references for the above indices were: FT3, 3.50–6.50 pmol/L; FT4, 11.50–23.50 pmol/L; TSH, 0.30–5.00 μIU/mL; Tg, 0–55.00 ng/mL; TgAb, 0-40.00 IU/mL. In the current study, TgAb > 40 IU/mL was defined as positive, otherwise negative.

### MK measurement

Fasting blood samples were obtained and centrifuged to collect serums, which were aliquoted, and stored at −80 °C until use. MK concentrations (reported as pg/mL) were measured in the following steps by using a commercial kit (DuoSet ELISA, R&D systems Inc., USA) in enzyme-linked immunosorbent assay methodology. Step 1, 100 μl of each serum sample or standard was incubated in 96-well microplate (pre-coated with goat anti-human MK antibody) for 2 hours at room temperature. Step 2, after washing three times, biotinylated goat anti-human MK antibody was added and incubated with captured MK for 2 hours at room temperature. Step 3, after another thrice washing, 100 μl aliquots of streptavidin-conjugated horseradish-peroxidase were added and allowed to react for 30 minutes in dark place. Step 4, after plate washing, substrate solutions (1:1 mixture of H_2_O_2_ and tetramethylbenzidine) were added to the wells (100 μl per well) for a 20-minute reaction. Step 5, 1 mol/L H_2_SO_4_ (stop solution) was added (50 μl per well), and the optical densities of the wells were measured at 450 nm with a Multiskan MS Plate Reader (Labsystems, Helsinki, Finland). Step 6, after creating a standard curve by a four parameter logistic curve-fit method, concentrations of samples were determined.

The normal distribution of MK concentration in healthy people was reported to range between 0 to 625 pg/ml (with the mean value of about 253 pg/ml)^19^, although the exact normal level of MK has not been unanimously determined. In our prior research, we found that the normal level of MK was 255.01 ± 126.78 pg/ml^23^.

### Statistics

All data were presented as mean ± standard deviation, which was statistically analyzed by SPSS 17.0 (SPSS Incorporated, Chicago, Illinois, USA) software. Differences of indices between two groups of patients were measured by independent samples *T* test. ROC curves were drawn and diagnostic efficacies were then determined. After the optimal cut-off MK value was selected, sensitivity, specificity, diagnostic accuracy, PPV (positive predictive value) and NPV (negative predictive value) for differential diagnosis were assessed. A P value not exceeding 0.05 was considered as statistically significant.

## Additional Information

**How to cite this article**: Jia, Q. *et al*. Serum midkine as a surrogate biomarker for metastatic prediction in differentiated thyroid cancer patients with positive thyroglobulin antibody. *Sci. Rep.* **7**, 43516; doi: 10.1038/srep43516 (2017).

**Publisher's note:** Springer Nature remains neutral with regard to jurisdictional claims in published maps and institutional affiliations.

## Figures and Tables

**Table 1:** Data comparisons in different groups of DTC* patients with positive TgAb* at the moment of their first ^131^I ablation.

Group^#^	Age	MK*	TgAb*	Tg*^@^	FT3*	FT4*	TSH*
(Case number)	(years old)	(255.01 ± 126.78 pg/ml^23^)	(0–40.00 IU/mL)	(0–55.00 ng/mL)	(3.50–6.50 mol/L)	(11.50–23.50 pmol/L)	(0.30–5.00 μIU/mL)
Group 1 (28)	47.43 ± 14.62	836.36 ± 395.10	299.26 ± 425.53	1.64 ± 2.76	1.95 ± 0.79	5.30 ± 1.98	88.25 ± 40.56
Group 2 (123)	47.39 ± 11.37	362.75 ± 246.45	238.60 ± 409.55	0.75 ± 2.20	2.07 ± 1.31	5.44 ± 3.36	94.62 ± 38.72
*T* value^	0.150	8.098	0.702	1.848	−0.474	−0.203	−0.779
*P* value^	0.988	<0.001	0.484	0.067	0.636	0.840	0.437

^#^Group 1 = ^131^I-avid metastases exist, group 2 = successful ablation without metastases.

*DTC = differentiated thyroid cancer, TgAb = thyroglobulin antibody, MK = midkine, Tg = thyroglobulin, FT3 = free triiodothyronine, FT4 = free thyroxine, TSH = thyroid stimulating hormone.

^analyzed by independent samples *T* test.

^@^Tg values should be considered not correct due to the presence of TgAb in this study.

**Table 2:** Prediction of whether ^131^I-avid metastases exist according to ROC curve-related data.

	MK*
Area under the curve	0.856
Optimal cut-off value	550 pg/ml
Sensitivity (%)	71%
Specificity (%)	85%
Accuracy (%)	83%
Positive predictive value (%)	54%
Negative predictive value (%)	93%

*MK = midkine.
